# Risk of betel chewing for oesophageal cancer in Taiwan

**DOI:** 10.1054/bjoc.2001.1927

**Published:** 2001-09

**Authors:** M-T Wu, Y-C Lee, C-J Chen, P-W Yang, C-J Lee, D-C Wu, H-K Hsu, C-K Ho, E-L Kao, J-M Lee

**Affiliations:** Division of Environmental Health and Occupational Medicine, National Health Research Institutes, Kaohsiung, Taiwan; Department of Occupational Medicine, Kaohsiung Medical University Hospital, Kaohsiung, Taiwan; Graduate Institute of Occupational Safety and Health, Kaohsiung Medical University, Taiwan; Department of Surgery, National Taiwan University Hospital, Taipei, Taiwan; Graduate Institute of Epidemiology, College of Public Health, National Taiwan University, Taipei, Taiwan; Department of Gastroenerology, Kaohsiung Medical University Hospital, Kaohsiung, Taiwan; Department of Chest Surgery, Kaohsiung Veterans General Hospital, Kaohsiung, Taiwan; Department of Chest Surgery, Kaohsiung Medical University Hospital, Kaohsiung, Taiwan

**Keywords:** oesophageal cancer, squamous-cell carcinoma, betel

## Abstract

Among 104 cases of squamous-cell oesophageal carcinoma patients and 277 controls in Taiwan, after adjusting for cigarette smoking, alcohol consumption, and other confounders, we found that subjects who chewed from 1 to 495 betel-year and more than 495 betel-years (about 20 betel quid per day for 20 years) had 3.6-fold (95% Cl = 1.3–10.1) and 9.2-fold risk (95% Cl = 1.8–46.7), respectively, of developing oesophageal cancer, compared to those who did not chew betel. © 2001 Cancer Research Campaign http://www.bjcancer.com


*British Journal of Cancer* (2001) **85**(5), 658–660
© 2001 Cancer Research Campaign
doi: 10.1054/ bjoc.2001.1927, available online at http://www.idealibrary.com on http://www.bjcancer.com

Short communication

### Risk of betel chewing for oesophageal cancer in Taiwan

**M-T Wu[1,2,3], Y-C Lee[4], C-J Chen[5], P-W Yang[4], C-J Lee[4], D-C Wu[6], H-K Hsu[7], C-K Ho[2,3], E-L Kao[8] and J-M Lee[4]**

[1]Division of Environmental Health and Occupational Medicine, National Health Research Institutes, Kaohsiung, Taiwan; [2]Department of Occupational Medicine, Kaohsiung Medical University Hospital, Kaohsiung, Taiwan; [3]Graduate Institute of Occupational Safety and Health, Kaohsiung Medical University, Taiwan; [4]Department of Surgery, National Taiwan University Hospital, Taipei, Taiwan; [5]Graduate Institute of Epidemiology, College of Public Health, National Taiwan University, Taipei, Taiwan; [6]Department of Gastroenerology, Kaohsiung Medical University Hospital, Kaohsiung, Taiwan; [7]Department of Chest Surgery, Kaohsiung Veterans General Hospital, Kaohsiung, Taiwan; [8]Department of Chest Surgery, Kaohsiung Medical University Hospital, Kaohsiung, Taiwan

**Summary** Among 104 cases of squamous-cell oesophageal carcinoma patients and 277 controls in Taiwan, after adjusting for cigarette smoking, alcohol consumption, and other confounders, we found that subjects who chewed from 1 to 495 betel-year and more than 495 betel-years (about 20 betel quid per day for 20 years) had 3.6-fold (95% Cl = 1.3–10.1) and 9.2-fold risk (95% Cl = 1.8–46.7), respectively, of developing oesophageal cancer, compared to those who did not chew betel. © 2001 Cancer Research Campaign http://www.bjcancer.com

**Keywords**: oesophageal cancer; squamous-cell carcinoma; betel

Oesophageal cancer is the 11th leading cause of cancer deaths in Taiwan, the 6th among males (DOH/ROC, 1999) and in 1999, the age-adjusted mortality rate was 3.93 per 100 000. Cigarette smoking and alcohol consumption are known to have important effects on this cancer (Pottern et al, 1981; Yu et al, 1988; Tavani et al, 1993).

Areca (betel nut) chewing, is a major addiction in Southeast Asia, especially in India and Taiwan. Although the effect of betel, with or without tobacco, on oesophageal cancer risk has been studied in India (Jussawalla, 1981; Nandakumar et al, 1996), this has not been done in Taiwan, where areca, or betel, is most often chewed with lime and *piper betle* influorescence (Chen et al, 1999). Because betel quid is not chewed with tobacco in Taiwan, it is possible to investigate the independent risks of betel, and cigarette tobacco and alcohol, on developing oesophageal cancer.

### MATERIALS AND METHODS

### Selection of cases and controls

Cases were histologically confirmed oesophageal squamous-cell carcinoma from the Department of Chest Surgery at National Taiwan University Hospital in Taipei, Taiwan. The Department of Preventive Medicine at this hospital chose 1 to 3 control subjects without malignancy matched on period of hospitalization age (± 3 y) and gender. In total, 104 cases (94 males and 10 females) and 277 controls (256 males and 21 females) were recruited for interview between July, 1996 and October 2000.

Subjects were interviewed by a trained interviewer using a standardized questionnaire. Informed consent was obtained from all subjects. Information on habitual substance use included whether the subject had ever been a habitual betel chewer, cigarette smoker, or alcoholic beverage drinker, what year the subject started and quit, the duration of consumption, the daily amount consumed, and type of alcoholic beverage consumed. Subjects who reported smoking more than 10 cigarettes per week for at least 6 months were defined as cigarette smokers, those who reported regularly chewing betel quid for at least 6 months were defined as betel chewers, and those who reported drinking beer, wine or distilled spirits more than once a week for at least 6 months were defined as alcoholic beverage drinkers.

*Received 15 December 2000*
*Revised 28 March 2001*
*Accepted 27 April 2001*

*Correspondence to:* M-T Wu (mingtsangwu@yahoo.com) and J-M Lee (ntuhlee@yahoo.com)

### Statistical analysis

Lifetime consumption of tobacco was calculated by multiplying the number of packs per day by the number of years smoked, giving pack-years. Lifetime consumption of betel quid was calculated as betel years by multiplying the average number of betel quid per day by the number of years chewed. Lifetime consumption of alcoholic beverage was calculated as gram years by multiplying the concentration of alcohol in the consumed beverage by the amount consumed per day by the number of years consumed.

The generalized additive model was used to adjust for other confounding factors without imposing a rigid parametric assumption about their dependence on risk, as in a previous study (Hastie and Tibshirani, 1990). Unconditional logistic regression was used to assess the association between case/control status and chewing betel nut and use of other substances. For substance use, we used no cigarette smoking, no betel chewing, or no alcohol consumption (each as defined above) as baselines and compared these baselines to lifetime consumption of the 3 substances and categorized the use into 2 groups based on the median. In addition, the synergistic or combined effect of these substances was also examined for significance. The data were analysed using the SAS and S-plus statistical packages (SAS, 1988; Hastie and Tibshirani, 1990).

### RESULTS

There were 271 pathology-proven cases of oesophageal squamous-cell carcinoma at National Taiwan University Hospital between July, 1996 and October, 2000. 46% (77/167) of the non-recruited patients were found to have distant metastasis in this study,

BJOC 01-1927 658-660 20/8/01 3:08 pm Page 659

**Table 1** The combined effect of substance use in oesophageal cancer with adjustments for other potential confounders

| Number of substance use | Cases *n* (%) | Controls *n* (%) | ORI | (95% CI) | OR2 | (95% CI) |
|---|---|---|---|---|---|---|
| 0 | 16 (15.4) | 140 (50.5) | 1.0 | | 1.0 | |
| 1 | 14 (13.5) | 91 (32.9) | 1.8 | (0.8, 4.2) | 1.5 | (0.6, 3.8) |
| 2 | 36 (34.6) | 37 (13.4) | 12.3 | (5.6, 27.2) | 12.4 | (5.1, 29.7) |
| 3 | 38 (36.5) | 9 (3.3) | 63.9 | (23.7, 171.9) | 39.2 | (13.2, 116.1) |

OR1: after adjusting for age (> 65 vs. ≤ 65 y) and gender. OR2: after adjusting for age (> 65 vs. ≤ 65 y), gender, education level, tea consumption, and intake of green vegetable.

whereas only 20% (21/104) of the recruited patients were found to have distant metastasis, a significant difference ($P < 0.001$).

The mean age range of the 104 oesophageal cancer patients and 277 controls were 39–84 (mean 60.6) and 38–81 (mean 62.6) years, respectively. Table 1 shows the odds ratios for the combined effect of these substances on oesophageal cancer after adjusting for other covariates. It was found that the higher the number of substances used concurrently, the higher the risk for oesophageal cancer. Compared to those who abstained from these substances, subjects who consumed 1, 2 and 3 substances concurrently had 1.8-, 12.3- and 63.9-fold risk of oesophageal cancer, respectively (95% CI = 0.8–4.2, 5.6–27.2 and 23.7–171.9) after adjusting for age and gender. The findings remained similar after further adjustment for educational levels, tea consumption and intake of green vegetables. However, we did not find any significant interaction or joint effect between any 2 of these 3 substances (data not shown).

The smoothing plots of age and lifetime consumption of the substances after adjusting for educational levels, tea consumption, and intake of green vegetables are presented in Figure 1. Because the risk of contracting oesophageal cancer increases after about age 65 years, the patients were categorized as 65 years or younger and over 65 years for subsequent analysis, the risk of developing oesophageal cancer in the latter group increasing markedly. The relationship between lifetime consumption of all 3 substances and oesophageal cancer risk also increased sharply. Although there were instances of higher dose users showing a small decline in risk, the observations were relatively few and insignificant. For example, only 8 and 7 observations in the groups of heavy smokers and heavy chewers had a relatively low risk for oesophageal cancer, but they still had a higher risk than those who did not smoke or chew (Figure 1).

We also examined the effect of lifetime consumption of the substances on oesophageal cancer risk. The median cut-off points for lifetime consumption of these substance were 30 pack-year for cigarette smokers, 495 betel-year for areca chewers, and 1220 gram-year for alcoholic beverage drinkers. The group that chewed 495 betel-year chewed an average of about 20 betel quid per day for 20 consecutive years. The group that consumed 1220 gram-year consumed an average of four 300–350 c.c. cans of beer (5% alcohol) per day for 20 consecutive years. In the final regression model, we found that education levels and substance use, including cigarette smoking, betel chewing, and alcohol consumption, were significant risk factors of oesophageal cancer (Table 2). After adjusting for other covariates, subjects who consumed 1–495 betel-year and more than 495 betel-years (about 20 betel quid per day for 20 years) were found to have a 3.6-fold (95% CI = 1.3–10.1) and a 9.2-fold (CI 1.8–46.7) risk, respectively, of developing oesophageal cancer, compared to those who did not. For cigarette smoking, subjects who smoked more than 30 pack-year had a 3.2-fold risk (95% CI = 1.5–6.8), compared to those who did not smoke. Compared to those who did not drink, subjects who

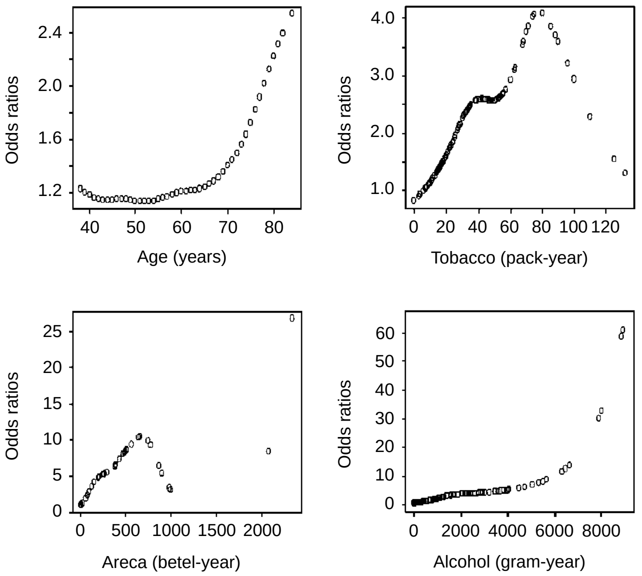

**Figure 1** Regression smoothing plots of oesophageal cancer with age or lifetime consumption of tobacco, areca, or alcohol, using generalized additive modelling approaches to adjust for gender, education level, tea consumption, and intake of green vegetables (6 observations of alcohol (gram-year) > 10 000 and odds ratios > 100 were discarded)

**Table 2** Predictors of oesophageal cancer with substance use and other covariates

| Variables | OR1 | (95% CI) | OR2 | (95% CI) |
|---|---|---|---|---|
| Age (in years) | | | | |
| > 65 vs. ≤ 65 | | | 1.0 | (0.5, 2.2) |
| Gender | | | | |
| Female vs. male | | | 2.1 | (0.7, 6.9) |
| Education levels (number of years) | | | | |
| 1–9 vs. none | 0.1 | (0.05, 0.4) | 0.2 | (0.08, 0.6) |
| ≥ 10 vs. none | 0.05 | (0.02, 0.1) | 0.09 | (0.03, 0.3) |
| Cigarette smoke (pack-year) | | | | |
| ≤ 30 vs. none | 1.6 | (0.7, 3.5) | 1.8 | (0.8, 4.3) |
| > 30 vs. none | 3.2 | (1.5, 6.8) | 3.7 | (1.6, 8.7) |
| Areca chewing (betel-year) | | | | |
| ≤ 495 vs. none | 3.6 | (1.3, 10.1) | 3.7 | (1.3, 10.9) |
| > 495 vs. none | 9.2 | (1.8, 46.7) | 9.4 | (1.8, 48.3) |
| Alcohol consumption (gram-year) | | | | |
| ≤ 1220 vs. none | 2.0 | (0.9, 4.6) | 2.2 | (0.9, 5.1) |
| > 1220 vs. none | 9.7 | (4.3, 22.0) | 9.8 | (4.2, 22.6) |
| Tea consumption (≥ 1 time per week) | | | | |
| Yes vs. no | | | 0.6 | (0.3, 1.3) |
| Intake of green vegetables (≥ 1 time per week) | | | | |
| Yes vs. no | | | 0.3 | (0.09, 1.2) |

*© 2001 Cancer Research Campaign* 

BJOC 01-1927 658-660 20/8/01 3:08 pm Page 660

consumed more than 1220 gram-year of alcohol (about 4 cans of beer per day for 20 years) in a lifetime had a 9.7-fold risk for oesophageal cancer (95% CI = 4.3–22.0). After further adjusting for age, gender, tea consumption and intake of green vegetables, these findings remained the same (Table 2).

### DISCUSSION

Besides cigarette smoking and alcoholic beverage consumption, betel chewing was found to be a major independent risk factor for oesophageal cancer, the more betel consumed during a lifetime, the higher the risk. Previous studies have found that chewing ‘pan’ (without tobacco), which consists betel leaf, betel nut and slaked lime, was a significant risk factor for oesophageal cancer in India (Jussawalla, 1981; Nandakumar et al, 1996). Our study confirmed this association in Taiwan, although the content of betel quid in India is a little different from that in Taiwan (Chen et al, 1999). Since most of the cases in this study were to evaluate possible surgery, it is likely that a smaller proportion of our cases had metastases than the non-recruited cases.

In Taiwan, *piper betle* influorescence, which contains about 15 mg $g^{-1}$ safrole is frequently added to betel quid (Chen et al, 1999; Liu et al, 2000). During chewing, the concentration of safrole can reach 420 μM. Animal experiments have found that DNA adducts can be formed in the livers of mice by 1′-hydroxysafrole, a metabolite of safrole (Borchert et al, 1973; Ioannides et al, 1981; Randerath et al, 1984; Reddy and Randerath, 1990). In addition, Ramchandani and his co-workers (1998) found that administration of 25 mg pan masala extract, a dry powder mixture of areca nut, catechu, lime, and some unspecific spices and flavouring agents, to diethylnitrosamine-initiated ICRC mice significantly enhanced the growth of oesophagus papilloma. These authors concluded that habitual use of pan-masala may exert a carcinogenic and co-carcinogenic influence in the stomach and oesophagus.

The prevalence of betel chewing in the Taiwanese population is over 10% (Ko et al, 1992). One study reporting an association between betel chewing and oral cancer (Ko et al, 1995) found that subjects who swallowed betel juice were at a significantly higher risk than those who did not. These workers therefore stressed the importance of examining the effect of betel chewing on cancer of the pharynx, larynx, oesophagus and stomach. Although we demonstrated that chewing betel quid can cause oesophageal cancer, we did not collect information on whether betel juice was swallowed. Another limitation is that we lack information on the type of substances consumed with betel nut. Although most Taiwanese people consume a combination of betel nut, *piper betle* influorescence and lime paste, some use betel nut wrapped in betel leaf. Betel leaf contains eugenol and hydroxycavicol, which are thought to be antimutagenic and anticarcinogenic (Bhide et al, 1991; Ko et al, 1995). The latter workers have reported that chewing betel nut wrapped in betel leaf seemed to be less of an oral cancer risk than the combination of betel nut, *piper betle* influorescence and lime paste.

We found a combined effect of consuming cigarettes, areca, and alcohol on oesophagus cancer risk, but not a significant joint or synergistic effect caused by any two of the substances: this might be due to our small sample size.

In conclusion, in addition to cigarette smoking and alcohol consumption, areca chewing is an independent potent risk factor for oesophagus cancer in Taiwan and also a combined effect of these 3 substances.

### ACKNOWLEDGEMENTS

This research was supported by a grant from National Science Council, Republic of China (Grant No. NSC 89-2314-B-037-113) and an award of the American Bureau for Medical Advancement in China, Inc. (ABMAC) for Ming-Tsang Wu.

### REFERENCES

Bhide SV, Zariwala MBA, Amonkar AJ and Azuine MA (1991) Chemopreventive efficacy of a betel leaf extract against benzo[*a*]pyrene-induced forestomach tumors in mice. *J Ethnopharmacol* **34**: 207–213

Borchert P, Wislocki PG, Miller JA and Miller EC (1973) The metabolism of the naturally occurring hepatocarcinogen safrole to 1′-hydroxysafrole and the electrophilic reactivity of 1′-acetoxysafrole. *Cancer Res* **33**: 575–589

Chen CL, Chi CW, Chang KW and Liu TY (1999) Safrole-like DNA in oral tissue from oral cancer patients with a betel quid chewing history. *Carcinogenesis* **20**: 2331–2334

Department of Health, Republic of China (1999) “Health Statistics: II. Vital Statistics.” Department of Health, Taipei.

Hastie TJ and Tibshirani RJ (1990) Generalized additive models. New York: Chapman & Hall

Ioannides C, Delaforge M and Parke DV (1981) Safrole: Its metabolism, carcinogenicity and interactions with cytochrome p-450. *Fd Cosmet Toxicol* **19**: 657–666

Jussawalla DJ (1981) Oesophageal cancer in India. *J Cancer Res Clin Oncol* **99**: 29–33

Ko YC, Chiang TA, Chang SJ and Hsief SF (1992) Prevalence of betel quid chewing habit in Taiwan and related socio-demographic factors. *J Oral Pathol Med* **21**: 261–264

Ko YC, Huang YL, Lee CH, Chen MJ, Lin LM and Tsai CC (1995) Betel quid chewing, cigarette smoking and alcohol consumption related to oral cancer in Taiwan. *J Oral Pathol Med* **24**: 450–453

Liu CJ, Chen CL, Chang KW, Chu CH and Liu TY (2000) Safrole in betel quid may be a risk factor for hepatocellular carcinoma: case report. *CMAJ* **162**: 359–360

Nandakumar A, Anantha N, Pattabhiraman V, Prabhakaran PS, Dhar M, Puttaswamy K, Venugopal TC, Reddy NM, Rajanna, Vinutha AT and Srinivas (1996) Importance of anatomical subsite in correlating risk factors in cancer of the oesophagus-report of a case-control study. *Br J Cancer* **73**: 1306–1311

Pottern LM, Morris LE, Blot WJ, et al. (1981) Esophageal cancer among Black men in Washington, DC. I. Alcohol, tobacco and other risk factors. *J Natl Cancer Inst* **67**: 777–783

Ramchandani AG, D’Souza AV, Borges AM and Bhisey RA (1998) Evaluation of carcinogenic/co-carcinogenic activity of a common chewing product, pan masala, in mouse skin, stomach and esophagus. *Int J Cancer* **75**: 225–232

Randerath K, Haglund RE, Phillips DH and Reddy MV (1984) $^{32}$P-post-labelling analysis of DNA adducts formed in the livers of animal treated with safrole, estragole and other naturally-occurring alkenylbenzenes. I. Adult female CD-1 mice. *Carcinogenesis* **5**: 1613–1622

Reddy MV and Randerath K (1990) A comparison of DNA adduct formation in white blood cells and internal organs of mice exposed to benzo[*a*]pyrene, dibenzo[*c,g*]carbazole, safrole and cigarette smoke condensate. *Mutat Res* **241**: 37–48

SAS Institute Inc. (1988) SAS® Language Guide for Personal Computers, Release 6.03 Edition. Cary, NC: SAS Institute Inc.

Tavani A, Negri E, Franceschi S and Vecchia CL (1993) Risk factors for esophageal cancer in women in Northern Italy. *Cancer* **72**: 2531–2536

Yu MC, Garabrant DH, Peters JM and Mack TM (1988) Tobacco, alcohol, diet, occupation and carcinoma of the esophagus. *Cancer Res* **48**: 3845–3848

 © 2001 Cancer Research Campaign

